# Supplementary material for: Accumulation of Proline in Plants under Contaminated Soils—Are We on the Same Page?
Source: Antioxidants (Basel). 2023 Mar 8;12(3):666. doi: 10.3390/antiox12030666 (PMC10045403; doi:10.3390/antiox12030666)
Supplement: Supplementary file 1 [file antioxidants-12-00666-s001.zip › antioxidants-2243653-supplementary.pdf]

Sofia Spormann[1,2]*, Pedro Nadais[2], Filipa Sousa[1,2], Mafalda Pinto[1,2], Maria Martins[1,2], Bruno Sousa[1,2], Cristiano Soares[1,2], Fernanda Fidalgo[1,2]

Supplementary Material

Table S1

Effects of applying stress mitigators to plants facing salinity-induced stress, namely on the endogenous levels of proline.

**Stress factor**		**Plant species**	**Stress-induced effects in comparison to the control**	**Mitigation treatment**	**Treatment-induced effects in comparison to the stress factor**	**Reference**
**Salinity**	200 mM NaCl	*Cucumis sativus*	↓ Chl and Car, ↓ g_s<br>↑ H_2O_2, MDA, and EL, ↑ phenols, ↓ RWC, ↑ **proline**<br>↑ SOD, CAT, GR, and APX, ↓ AsA, ↑ ABA, JA, and SA, ↑ Na and ↓ K, Ca, Mg, Fe, Zn, Mn, and Cu levels	Inoculation with arbuscular mycorrhizal fungi	↑ Chl and Car, ↑ g_s<br>↓ H_2O_2, MDA, and EL, ↑ phenols, ↑ RWC, ↑ **proline**<br>↑ SOD, CAT, GR, and APX, ↑ AsA content, ↓ ABA and ↑ JA, and SA, ↓ Na and ↑ K, Ca, Mg, Fe, Zn, Mn, and Cu levels	[185]
	150 mM NaCl	*Vicia faba*	↓ fresh and dry biomass<br>↓ yield, ↓ No. of nodules<br>↓ leaf area, ↓ soluble sugars and soluble proteins<br>↑ free amino acids<br>↑ **proline,** ↑ Na and ↓ K, Ca, and Mg<br>↑ MDA, ↑ SOD, CAT, POX, and APX	Auxin<br>(1.5 mM Indole-3-acetic acid)<br><br>CK<br>(0.9 mM 6-Benzylaminpurine)	↑ fresh and dry biomass<br>↑ yield, ↑ No. of nodules<br>↑ leaf area, ↑ soluble sugars and soluble proteins<br>↑ Free amino acids<br>↓ **proline,** ↓ Na and ↑ K, Ca, and Mg<br>↓ MDA, ↑ SOD, CAT, POX, and APX	[186]
	150 mM NaCl	*Brassica juncea*	↓ length and fresh and dry biomass, ↓ leaf area<br>↓ Chl, ↓ photosynthetic rate, g_s and WUE, ↑ SOD, CAT, and POX, ↑ **proline**	Si<br><br>BRs<br>(1 x 10^{-8} mM epibrassinolide)<br><br>Si and BRs	↑ length and fresh and dry biomass, ↑ leaf area<br>↑ Chl, ↑ photosynthetic rate, g_s, and WUE, ↑ SOD, CAT, and POX, ↑ **proline (BRs and combination of Si and BRs)**<br>**= proline (Si)**	[187]
	80 mM NaCl	*Cicer arietum*	↓ dry biomass<br>↓ No. of flowers, pods, and nodules, ↓ Chl and Car, ↓ leghemoglobin and ↑ lignin, ↑ ROS and MDA content, ↑ SOD, CAT, and POX, ↓ protein,<br>↑ phenols and soluble sugar,<br>↑ **proline**	SA<br>(0.5 mM)	↑ dry biomass<br>↑ = No. of flowers and pods and ↑ No. of nodules, ↑ Chl and Car,<br>↑ leghemoglobin and ↑ lignin<br>↓ ROS and MDA content<br>↑ SOD, CAT, and POX,<br>↑ protein, phenols, and soluble sugars, ↑ **proline**	[188]
	200 mM NaCl	*Zea mays*	↓ diameter, length and fresh and dry biomass, ↓ leaf area, ↓ Chl and Car,<br>↓ photosynthetic rate, g_s, and *E*, ↓ RWC, ↑ soluble sugars, ↑ EL, ROS and MDA, = SOD and POX, ↑ CAT and APX, ↑ **proline**	Melatonin<br>(30, 60 and 90 µM)	↑ diameter, length and fresh and dry biomass (except = root fresh weight)<br>↑ leaf area, ↑ Chl and Car,<br>↑ photosynthetic rate, g_s, and *E*<br>↑ RWC, ↓ soluble sugars,	[189]

					↓ EL and ROS and MDA, ↑ SOD, POX, CAT, and APX, ↓ **proline**	
	50-200 mM NaCl	*Setaria italica*	↓ growth, ↑ H_2O_2 content ↑ soluble sugars, GB, protein and GSH, ↑ SOD, CAT, GR, GST, POX, and APX, ↑ **proline**	Se (1 µM)	↑ growth, ↓ H_2O_2 content ↑ soluble sugars, GB, protein and GSH, ↑ SOD, CAT, GR, GST, POX, and APX, ↑ **proline**	[190]
		Panicum miliaceum				
	50 mM NaCl	*Cucumis sativus*	↓ growth, ↑ ROS, ↑ **proline,** ↑ P5CS and ↓ ProDH and OAT activity, ↑ ABA (↓ at 12 d), IAA (↓ at 12 d) and CK (↓ at 6 d) and ↓ SA	Si (0.3 mM Na_2SiO_3)	↑ growth, ↓ ROS, ↑ **proline (↓ at 9 d),** ↑ P5CS (↓ at 9 d) and OAT and ↓ ProDH (↑ at 6 d) ↓ ABA (↑ at 12 d), IAA (↑ at 9 d) and CK (↑ at 6 d) and ↑ SA, (remained constant in NaCl + Si, but decreased overtime in NaCl)	[50]
	100 mM NaCl	*Sorghum bicolor*	↓ RWC, leaf area, and dry biomass, ↓ SOD, CAT, and APX activity, ↑ **proline,** ↑ MDA ↑ Na, ↓ K	Si (28.6 mM via foliar spray, irrigation, or both)	↑ RWC (= in foliar spray) leaf area (= in foliar spray), and dry biomass, ↑ SOD, CAT, and APX activity, ↓ **proline (= for foliar spray),** ↓ MDA, ↓ Na and ↑ K	[191]
		Helianthus annus			*= RWC (↑ in the combination) ↑ leaf area and dry biomass ↑ SOD, CAT, and APX activity ↑ **proline,** ↓ MDA, ↓ Na, ↑ K	

↑, ↓, and = symbolize the reported increase, decrease or maintenance of a certain parameter, respectively.
ABA, abscisic acid; APX, ascorbate peroxidase; AsA, ascorbate; Asp, aspargine; BR, brassinosteroids; CA, carbonic anhydrase; Car, carotenoids; CAT, catalase; Chl, chlorophylls; *Ci*, intercellular CO_2 concentration; CK, cytokinins; DHAR, dehydroascorbate reductase; DPPH, 2,2-diphenyl-1-picrylhydrazyl; *E*, transpiration rate; EL, electrolyte leakage; Fv/Fm, maximum quantum yield of PSII; GA_3, gibberellic acid; GB, glycine betaine; GDH, glutamate dehydrogenase**;** Glu, glutamate; Gly I, glyoxalase I; Gly II, glyoxalase II; GOGAT**,** glutamate synthase; GPX, glutathione peroxidase; GR, glutathione reductase; g_s stomatal conductance; GS, glutamine synthetase; GSH, glutathione; GSSG, glutathione disulfide; GST, glutathione S-transferase**;** GST, glutathione S-transferases; H_2O_2, hydrogen peroxide; IAA: indole acetic acid; JA, jasmonic acid; LP, lipid peroxidation; MDA, malondialdehyde; MDHAR, monodehydroascorbate reductase; NADPH, nicotinamide adenine dinucleotide phosphate; NPQ, non-photochemical quenching; NR, nitrate reductase; $O_2^{\cdot -}$, superoxide anion; OAT, ornithine aminotransferase; P5C, pyrroline-5-carboxylate; P5CS, pyrroline-5-carboxylate synthase; P_N, net photosynthetic rate; POX, peroxidase; PPO, polyphenol oxidase**;** ProDH, proline dehydrogenase; PTS, trisodium-8-hydroxy-1,3,6-pyrenetrisulphonic acid; Put, putrescine; ROS, reactive oxygen species; RuBisCO, ribulose-1,5-bisphosphate carboxylase-oxygenase; RWC, relative water content; SA, salicylic acid; SAMDC, S-adenosylmethionine decarboxylase; SOD, superoxide dismutase; Spd, spermidine; Spm, spermine; TAC, total antioxidant capacity; WUE, water use efficiency; Φ PSII, quantum yield of PSII.

Table S2

Physiological effects observed in plants under abiotic stress caused by exposure to contaminant levels of salt, heavy metals, and xenobiotics and in response to the exogenous application of proline.

Stress factor		Plant species	Stress-induced effects in comparison to the control	Proline treatment	Pro-induced effects in comparison to stress	Citation
**Salinity**	100 - 200 mM	*Hordeum vulgare* L.	↓ growth**,** ↑ **proline**, ↑ LP, ↑ SOD, CAT, APX, GR and POX, = DHAR	5 mM	= growth, ↑ **proline,** ↓ LP, ↓ SOD, ↑ CAT, GR and POX, = APX and DHAR	[88]
	100 mM	*Cucumis sativus* L.	↓ growth, = **proline**, ↓ leaf RWC, ↑ Na^+/K^+, ↑ Cl^-, ↑ MDA, ↑ SOD, CAT APX, =POD	10 mM	↑ growth, ↑ **proline**, ↑ leaf RWC, ↓ Na^+/K^+, = Cl^-, ↓ MDA, ↓ SOD, ↑ POX, =CAT and APX	[81]
	25 mM	*Oriza sativa L.*	↑ PTS uptake, ↑ Na^+, = K^+, = **proline** and betaine	1 and 5 mM	↓ PTS uptake, ↓ Na^+, ↑ K^+, ↑ **proline** and = betaine	[192]
	30 mM	*Vigna radiata* L.	↓ GSH and GSSG, ↑ GPX, GR, GST and Gly, ↑ H_2O_2, ↑ MDA	15 mM	↑ GSH, ↓ GSSG, = GPX, GST and Gly, ↓ GR, ↓ H_2O_2, ↓ MDA	[89]
	200 and 100 mM	*Olea europaea* L. cv. Chemlal	↓ growth, ↓ leaf water relations, ↓ photosynthetic activity, ↓ photosynthetic pigments, ↑ **proline**, ↑ sugars, ↑ SOD, CAT, APX and PPO	25 and 50 mM	↑ growth, ↑ leaf water relations, ↑ photosynthetic activity, ↑ photosynthetic pigments, ↑ **proline**, ↓ sugars, = SOD, ↑ CAT and APX, ↓ PPO	[91]
	100 mM	*Sorghum bicolor* L.	↓ germination rate, ↓ growth, ↓ Chl	0,5 and 100 mM	↑ germination rate, ↑ growth, ↑ Chl	[193]
	200 mM	*Vigna radiata* L.	↓ AsA, ↑ GSH and GSSG, ↑ H_2O_2, ↑ LP, ↓ MDHAR, DHAR, CAT and Gly I, ↑ GST, GPX and Gly II, = APX and GR	5 mM	↑ AsA, ↑GSH and GSSG, ↓ H_2O_2, ↓ LP, = MDHAR e GST, ↑ DHAR, CAT, Gly I, GPX and Gly II, APX and GR	[89]
	0 - 400 mM	*Oriza sativa* L.	↓ germination rate, ↓ growth, ↓ photosynthetic rate and Chl, ↑ **proline**, ↓ proteins	1 mM	↑ germination rate, ↑growth, ↑ photosynthetic rate and Chl, = **proline**, ↑ proteins	[194]
	EC = 1.5 – 13.5 dS m^{-1}	*Brassica napus* L.	↓ growth, ↓ germination rate, ↓ photosynthetic pigments, ↑ **proline**, ↑ sugars, ↑ AsA, ↑ Na^+ and Cl^-, ↓ N, P and K^+	10 mM	↑ growth, ↑ germination rate, ↑ photosynthetic pigments, ↑ **proline**, ↑ sugars, = AsA, ↑ Na^+, Cl^- and K^+, ↑ N, P	[195]
	100 mM	*Oriza sativa* L.	↓ growth, ↓ Chl, ↑ PTS uptake, ↑ Na^+/K^+	1 mM	↑ growth, ↑ Chl, ↓ PTS uptake, ↓ Na^+/K^+	[196]
	100 mM	*Oriza sativa* L.	↓ growth, ↑ Na^+/K^+, ↑ **proline**, ↑ H_2O_2, ↑ soluble sugars, ↑ H_2O_2, ↑ SOD, POX, APX and CAT, ↑ P5CS and P5CR expression ↑ *Cu/Zn SOD, Mn SOD, cAPX* and *CAT* expression	10 mM	= growth, ↓ Na^+/K^+, ↑ **proline**, ↑ H_2O_2, ↓ SOD, POX and CAT, = APX, ↑ P5CS and P5CR expression, = *Cu/Zn SOD, Mn SOD, cAPX* and *CAT* expression	[197]

	200 mM	*Oriza sativa* L.	↓ growth, ↑ Na^+/K^+, ↑ SOD, POX, APX and CAT, ↑ H_2O_2	10 mM	↓ growth, ↓ Na^+/K^+, ↓ SOD, POX, ↑ APX and CAT, ↓ H_2O_2	[198,201]
	EC = 1.84 – 8.97 dS m^{-1}	*Phaseolus vulgaris* L.	↓ growth, ↓ photosynthetic pigments, ↑ **proline**, ↑ AsA, ↑ NO_3^- and NO_2^-, ↓ P and K^+ ↑ Na^+, ↑ Na^+/K^+, ↑ SOD, CAT and POD	5 mM	↑ growth, ↑ photosynthetic pigments, ↑ **proline**, ↑ AsA ,↓ NO_3^- and NO_2^-, ↑ P and K^+, ↓ Na^+, ↓ Na^+/K^+, ↑ SOD, CAT and POD	[71]
	100 – 200 mM	*Hordeum vulgare* L.	↓ growth, ↓ photosynthetic pigments, ↑ **proline**, ↓ RWC, ↓ sugars, ↑ EL, ↑ SOD, POX and CAT	1 mM	↑ growth, ↑ photosynthetic pigments, ↑ **proline**, ↑ RWC, ↑ sugars, ↑ EL, =SOD, ↑ POX and CAT	[90]
	100 mM	*Oryza sativa* L.	↓ germination rate, ↓ relative germination energy, ↓ α and β - amylase activity	4 - 45 mM	↑ germination rate, ↑ relative germination energy, ↑ α and β - amylase activity	[199]
	100 mM	*Pisum sativum* L.	↓ growth ↓ Chl, ↓ C_i and g_s, ↓ RWC, ↓ photosynthetic activity, ↑ $O_2^{\cdot-}$ and H_2O_2 , ↑ LP, ↓ membrane stability, ↑ EL, ↑ arginine decarboxylase and ornithine decarboxylase, ↑ S-adenosylmethionine, ↑ polyamines, ↑ **proline** and GB, ↑ total amino acids, sugars phenols and tocopherols	60 mM	↑ growth, ↑ Chl ↑ C_i, = g_s, ↑ RWC, = photosynthetic activity, ↓ $O_2^{\cdot-}$ and H_2O_2, ↓ LP, ↑ membrane stability, ↓ EL, ↑ arginine decarboxylase and ornithine decarboxylase, ↑ S-adenosylmethionine, ↑ polyamines, ↑ **proline** = GB, ↑ total amino acids, sugars phenols and tocopherols	[83]
	150 and 300 mM	*Oryza sativa* L.	↓ RWC, ↓ Chl, ↑ LP, ↑ **proline**, ↑ linoleate 13s-, LOX, ↑ H_2O_2, ↓ AsA and GSH, ↑ GSSG, ↓ SOD, MDHAR, DHAR, CAT, GR and GPX, ↑ APX and GST	5 mM	↑ RWC, ↑ Chl, ↓ LP, ↑ **proline**, ↓ LOX, ↓ H_2O_2, ↑ AsA and GSH, ↓ GSSG, ↑ SOD, MDHAR, DHAR, CAT, GR, GPX, APX and GST	[84]
	100 mM	*Saccharum offcinarum* L.	↓ membrane integrity, = **proline**, ↓ proteins, ↑ Na^+, ↓ K^+, ↑ Na^+/K^+, ↑ POX, CAT and APX	20 mM	= membrane integrity, ↑ **proline**, = proteins, ↓ Na^+, = K^+ and Na^+/K^+, ↑ POX, CAT, = APX	[78]
	15 mM	*Glycine max* L. Merrill	↓ No. of pods and branches, ↓ seed weight and yield, ↓ seed protein and oil	25 mM	↑ No. of pods and branches, ↑ seed weight and yield, ↑ seed protein and oil	[200]
	150 mM	*Oryza sativa* L.	↓ growth, ↓ Chl, = LP, ↑ **proline**	5 – 15 mM	↑ growth, ↑ Chl, = LP, ↑ **proline**	[201]
	200 mM	*Eurya emarginata* (Thunb.)	↓ growth, ↓ RWC, ↑ LP, ↑ **proline**, ↑ Na^+/K^+, ↓ H^+-ATPase, CAT, POX, GPX and PDH, ↑ SOD and P5CS	10 mM	↑ growth, ↑ RWC, ↓ LP, ↑ **proline**, = Na^+/K^+, ↑ H^+-ATPase, SOD, CAT POX, ↓ P5CS, = PDH and GPX	[87]

	25 mM	*Zea mays* L.	↓ growth, ↑ Na^+/K^+, ↓ Chl, ↑ straw N, = straw P and S, ↓ grain N, P and S	25 - 100 mM	↑ growth, ↓ Na^+/K^+, = Chl, ↑ straw N, P and S, ↑ grain N, P and S	[92]
	25 – 100 mM	*Oryza sativa* L.	↓ growth, ↓ Chl, ↑ **proline**, ↓ AsA, ↓ POX and APX, = CAT, ↑ Na^+/K^+	25 and 50 mM	↑ growth, ↑Chl, ↑ **proline**, ↑ AsA, ↑ POX and APX, = CAT, ↓ Na^+/K^+	[72]
	200 and 400 mM	*Cakile maritima* L.	↓ relative growth rate, ↓ g_s, and photosynthetic rate and *E*, ↓ Fv/Fm, ↓ ΦPSII, ↑ NPQ, ↓ ETR, ↑ LP, ↓ RWC, ↓ osmotic potential (OP), ↑ Na^+ and Cl^-, = K^+, ↑ **proline** and soluble carbohydrates	20 mM	↑ relative growth rate, ↑ g_s, and photosynthetic rate and *E*, = Fv/Fm, ↑ ΦPSII, ↓ NPQ, ↑ ETR, ↓ LP, ↑ RWC, ↓ OP, ↑ Na^+ and Cl^-, ↑ K^+, ↑ **proline** and soluble carbohydrates	[80]
	EC = 2.8 – 5.6 dS m^{-1}	*Brassica juncea* L.	↓ growth, ↓ leaf water potential, ↑ EL, ↓ carbonic anhydrase, ↓ photosynthetic rate and relative attributes, ↑ Fv/Fm, ↑ CAT, POX and SOD, ↑ **proline**, ↓ yield	20 mM	↑ growth, ↑ leaf water potential, ↓ EL, ↑ carbonic anhydrase, ↑ photosynthetic rate and relative attributes, ↑ Fv/Fm, ↑ CAT, POX and SOD, ↓ **proline**, ↑ yield	[85]
	100 mM	*Zea mays L.*	↓ growth, ↑ **proline**, ↓ RWP, ↓ membrane stability, ↑ EL, ↑ SOD and GPOX, ↓ protein, ↑ IAA and ABA, ↓ GA and zeatin	0.5 mM	↑ growth, ↑ **proline**, ↑ RWP, ↑ MSI, ↓ EL, ↑ SOD and GP, ↑ protein, ↑ IAA, GA and zeatin, ↓ ABA	[202]
	60 and 120 mM	*Triticum aestivum* L.	↓ growth, ↓ Chl, ↑ sugars and phenols, ↑ **proline** and GB, ↑ Na^+, ↓ K^+, ↑ Na^+/K^+	50 and 100 mM	↑ growth, ↑ Chl, ↑ sugars and phenols, ↑ **proline** and GB, ↓ Na^+, ↑ K^+, ↓ Na^+/K^+	[77]
	25 mM	*Oryza sativa* L.	↑ H_2O_2, ↑ LP, ↓ *E*	1 mM	↓ H_2O_2, ↓ LP, ↑ *E*	[203]
	15 mM	*Glycine max* (L.) Merrill	↓ nodule number and activity, ↓ N fixation	25 mM	↑ nodule number and activity, ↑ N fixation	[93]
	25 – 100 mM	*Oryza sativa* L.	↓ growth, ↓ Chl, ↑ **proline**, ↓ AsA, ↓ POX and APX, = CAT, ↑ Na^+/K^+	25 and 50 mM	↑ growth, ↑Chl, ↑ **proline**, ↑ AsA, ↑ POX and APX, = CAT, ↓ Na^+/K^+	[72]
	25 and 100	*Onobrychis viciifolia L.*	↓ growth, ↓ RWC, ↑ LP, ↑ relative membrane permeability, ↑ **proline**, ↑ Na^+/K^+	2.5 mM	↑ growth, ↑ RWC, ↓ LP, ↓ relative membrane permeability, ↑ **proline**, ↓ Na^+/K^+	[79]
	80 mM	*Zea mays L.*	↓ growth, ↓ protein, = N compounds, ↑ Na^+ and Cl^-, ↓ K^+, ↑ Na^+/K^+	30 mM	↑ growth, ↑ protein, = N compounds, ↓ Na^+ and Cl^-, ↑ K^+, = Na^+/K^+	[74]
	50 – 200 mM	*Oryza sativa* L.	↓ germination rate, ↓ seed vigor index, ↓ α-amylase activity	1 - 10 mM	↑ germination rate, ↑ seed vigor index, ↑ α-amylase activity	[204]
	150 mM	*Triticum aestivum* L.	↓ growth, ↓ Chl, ↑ SOD, = CAT and POD	4 and 8 mM	↑ growth, ↑ Chl, ↑ SOD and CAT, = POD	[205]

	70 mM	*Sorghum bicolor* L.	↓ growth, ↑ membrane damage, ↓ RWC, ↓ photosynthetic rate and g_s, ↓ *E* and C_i, ↑ Na^+ and Cl^-, ↓ K^+ and Ca^+, ↑ Na^+/K^+, ↓sugars, = N compounds, ↓ Glu, GSH and Asp, ↑ aspartate and **proline**	30 mM	↑ growth, = membrane damage, = RWC, = photosynthetic rate and g_s, = *E* and C_i, ↓ Na^+ and Cl^-, ↑ K^+ and Ca^+, ↓ Na^+/K^+, =sugars and = N compounds, ↑ Glu, GSH, Asp, aspartate, and **proline**	[73]
**Heavy metals**	Cd 5-100 µM	*Solanum nigrum* L.	↓ growth, ↑ SOD and CAT, ↑ ROS, = GSH, ↑ membrane damage	1 mM	↑ growth, ↑ Cd uptake, ↑ SOD and CAT, ↓ ROS, ↑ GSH, ↓ membrane damage	[137]
	Cd 100 µM	*Nicotiana tabacum* L.	↓ growth, ↑ **proline**, ↑ LP, ↓ CAT, ↓ SOD, ↑ POX	1-10 mM	↑ growth, ↓ Cd uptake, ↓ LP, ↑ CAT, ↑ SOD, ↓ POX	[206]
	Cd 1 mM	*Vigna radiata* L.	↓ AsA, ↑ GSH, ↑ GSSG, ↑APX, ↓ DHAR, ↓ MDHAR, ↓ GR, ↑GPX, ↑GST, ↓ CAT, ↑ Glyoxalase I, ↓ Glyoxalase II, ↑ LP, ↑ H_2O_2	5 mM	↑ AsA, ↑ GSH, ↓ GSSG, ↑ APX, ↑ DHAR, ↑ MDHAR, ↑GR, ↑ GPX, ↑ GST, ↑ CAT, ↑ Glyoxalase I, ↑ Glyoxalase II, ↓ LP, ↓ H_2O_2	[89]
	Cd 1 mM	*Triticum aestivum* L.	↓ growth, ↓ photosynthetic pigments, ↑ **proline**, ↑ AsA, ↓ SOD, ↑ POX, ↑ LP, ↑ membrane permeability, ↑ Cu uptake, ↓ Zn uptake	1-10 mM	↑ growth, ↑ photosynthetic pigments, ↑ AsA, ↑ SOD, ↓ POX, ↓ LP, ↓ membrane permeability, ↓ Cd^{2+} uptake, ↓ Cu^{2+} uptake, ↑ Zn^{2+} uptake	[207]
	Cd 25-100 mg kg^{-1}	*Cicer arietinum* L.	-	20 mM	↑ growth, ↑ CA, ↑ *gS*, ↑ *Ci*, ↑ *E*, ↑ WUE, ↑ P_N, ↑ leaf water potential, ↑ POX, SOD, and CAT, ↑ seed proteins, ↑ seed yield	[119]
	Cd 200 µM	*Triticum aestivum* L.	↓ growth, ↓ Chl, ↓ leaf phenolics, ↑ LP, ↑ H_2O_2	20 mM	↑ growth, ↑ Chl b, ↑ leaf phenolics, ↓ LP, ↓ H_2O_2	[208]
	Cd 10-30 mg kg^{-1}	*Phoenix dactylifera* L.	↓ growth, ↓ macronutrients uptake, ↓ membrane stability, ↓ POX, ↑ APX, ↑ H_2O_2, ↓ starch, ↑ soluble sugars, ↑ total polyphenols, = Car	20 mM	↑ growth, ↑ macronutrient contents, ↓ Cd^{2+} uptake, ↑ membrane stability, ↑ POX, ↑ APX, ↓ H_2O_2, ↑ soluble sugars, ↑ total polyphenols, ↑ Car	[131]
	Cd 10-30 mg kg^{-1}	*Olea europaea* L.	↓ growth, ↓ Chl, ↓ Ca^{2+} uptake, ↓ olive oil content, ↓ P_N, ↓ *gS*, ↓ *E*, ↑ H_2O_2, ↑ LP, ↑ EL, ↑ **proline** ↑ SOD, CAT, GPX, and APX	10-20 mM	↑ growth, ↑ Chl, ↓ Cd^{2+} uptake, ↑ K^+ and Mg^{2+} uptake, ↑ olive oil content, ↑ P_N, ↑ *gS*, = *E*, ↓ H_2O_2, ↓ LP, ↓ EL, ↑ SOD, CAT, GPX, and APX	[209]

	Cd<br>25-100 mg kg^{-1}	*Cicer arietinum* L.	-	20 mM	↑ nodule growth, ↑ nodule leghemoglobin, ↑ nodule nitrogenase activity, ↑ nodule nitrogen, nodule carbohydrates, ↑ nodule GS, ↑ GOGAT and GDH, ↑ root nitrate, ↑ leaf nitrogen, ↑ leaf NR	[210]
	Cd<br>50 mg L^{-1}	*Triticum aestivum* L.	↑ growth, ↓ leaf water content, ↑ **proline**, ↑ LP, ↓ SOD	5-20 mM	↑ growth (only at 20 mM **proline** for 72h), ↑ leaf water content, ↓ LP, = SOD	[211]
	Cd<br>1.5 mM	*Phaseolus vulgaris* L.	↓ growth, ↓ yield, ↓ Chl, ↓ Car, ↑ **proline**, ↑ soluble sugars, ↑ GSH, ↓ RWC, ↓ membrane stability, ↑ LP, ↑ EL, ↓ P_N, ↓ *E*, ↑ CAT, POX, APX, SOD, and GR, ↓ N, P, and K^+ uptake, ↓ K^+/ Na^+	6 mM	↑ growth, ↑ yield, ↑ Chl, ↑ Car, ↑ RWC, ↑ membrane stability, ↓ LP, ↓ EL, ↑ P_N, ↑ *E*, ↓ Cd^{2+} uptake, ↑ CAT, APX, and SOD, = POX and GR, ↑ N and P uptake, ↑ K^+/ Na^+	[149]
	Cd<br>80 µM	*Brassica juncea* L.	↓ growth, ↓ Chl, ↓ Car, ↑ **proline**, ↓ P_N, ↓ *gS*, ↑ *Ci*, ↓ *E*, ↓ cell viability, ↑ H_2O_2, ↑ O_2^-, ↑ LP, ↑ CAT, ↑ leaf POX, = APX and SOD, ↓ GSH/GSSG	20-60 mg L^{-1}	↓ growth, ↑ Chl, ↑ Car, ↑ P_N, ↑ *gS*, ↓ *Ci*, ↑ *E*, ↓ cell viability, ↓ Cd^{2+} uptake, ↓ H_2O_2, ↓ O_2^-, ↓ LP, ↑ GSH/GSSG, ↑ SOD, POX, APX, CAT	[139]
	Cd<br>4-8 mg mL^{-1}	*Cajanus cajan* L.	↓ growth, ↓ Chl, ↓ Mg^{2+}, K^+ and Ca^{2+} uptake, ↑ H_2O_2, ↑ LP, ↓ P_N, ↓ *gS*, ↓ *E*, ↑ EL, ↑ **proline**, ↑ SOD, CAT, GPX, and APX	3 - 6 mM	↑ growth, ↑ Chl, ↑ Mg^{2+}, K^+, Ca^{2+} uptake, ↓ Cd^{2+} uptake, ↓ H_2O_2, ↓ LP, ↑ P_N, ↑ *gS*, ↑ *E*, ↓ EL, ↑ SOD, CAT, GPX, and APX	[144]
	Cu<br>25 µM	*Cicer arietinum* L.	↑ LP, ↓ Chl, ↑ **proline** (mainly in tolerant genotypes), ↑ phytochelatin-SH, = total acid-soluble SH, = GSH, ↑ SOD and CAT, ↓ APX	1 mM	↓ LP (in tolerant genotypes), ↑ tolerance index, = proteins	[212]
	Cu<br>400 µM	*Triticum aestivum* L.	↓ growth, ↓ yield, ↓ Chl, ↑ total soluble proteins, ↑ **proline**, ↓ P_N, ↓ *Ci*, ↓ *gS*, ↓ water-use-efficiency, ↑ NPQ, ↓ photochemical quenching, ↓ Electron transport rate, ↓ productivity of PSII, ↑ SOD, CAT, POX, ↓ K^+, Mg^{2+}, and Ca^{2+} uptake	80 mM	↑ growth, ↑ yield, ↑ Chl, ↑ P_N, ↑ *Ci*, ↑ g_S, ↑ water-use-efficiency, ↓ NPQ, ↑ photochemical quenching, ↑ electron transport rate, ↑ productivity of PSII, ↑ SOD, CAT, POD ↓ Cu^{2+} uptake, ↑ K^+, Mg^{2+}, and Ca^{2+} uptake	[130]

	Cr (VI) 0.6-20 mg L^{-1}	*Oryza sativa L.*	↓ growth, ↑ root cell death, ↑ LP, ↓ root length, area, volume, and diameter, ↑ root tip and branch number	1 μM	= growth, = root phenotype traits, ↓ root cell death, ↑ Cr(VI) uptake, ↓ LP	[137]
	Hg 0.2 mM	*Oryza sativa* L.	↑ H_2O_2, ↑ O_2^{-}, ↑ LP, ↓ GSH, ↑ GSSG, ↓ Chl, ↑ **proline**	2 mM	↓ H_2O_2, ↓ O_2^{-}, ↓ LP, ↓ Hg^{2+} uptake	[132]
	Hg 0.1-0.5 mM	*Coriandrum sativum* L.	↑ LP, ↓ Chl, ↓ Car, ↓ anthocyanins, ↑ **proline** ↑ GB, ↓ protein, ↑ CAT and DHAR, ↓ SOD and APX, ↓ AsA (at 0.5 mM Hg), ↓ GSH (up to 0.3 mM Hg), ↓ phenolic compounds	50 mM	↓ LP, ↑ Chl, ↑ Car, ↑ anthocyanins, ↑ GB, ↑ protein, ↑ SOD, APX, and DHAR, ↓ CAT, ↑ GSH (at 0.5 mM Hg), ↑ phenolic compounds	[147]
	Ni 200 μM	*Atropa belladonna* L.	↓ growth, ↑ LP, ↓ Put, ↑ Spm and Spd, ↑ oxidative degradation of Spm and Spd, ↑ endogenous Pro	5 mM	↓ Ni^{2+} uptake, ↓ LP, ↑ leaf water status, ↑ Fe uptake, ↓ oxidative degradation of Spm and Spd	[213]
	Ni 100 μM	*Pisum sativum* L.	↓ growth, ↓ Chl, ↓ photosynthetic activity, ↓ g_S, ↓ *Ci*, ↓ RWC, ↓ membrane stability, ↑ H_2O_2, ↑ O_2^{-}, ↑ LP, ↑ EL, ↑ polyamines, ↑ ADC, ↑ ODC, ↑ SAMDC, ↑ **proline** ↑ GB, ↑ free amino acids, ↑ soluble sugars, ↑ phenolic compounds, ↑ tocopherols	60 mM	↑ growth, ↑ Chl, ↑ photosynthetic activity, ↑ *gS*, ↑ *Ci*, ↑ RWC, ↑ membrane stability, ↓ H_2O_2, ↓ O_2^{-}, ↓ LP, ↓ EL, ↑ polyamines, ↑ ADC, ↑ ODC, ↑ SAMDC, = GB, ↑ free amino acids, ↑ soluble sugars, ↑ phenolic compounds, ↑ tocopherols,	[83]
	Ni 100 μM	*Pisum sativum L.*	↓ growth, ↓ Chl, ↓ photosynthetic activity, ↓ *gS*, ↓ *Ci*, ↓ number of stomata, ↓ stomatal size, ↓ WUE, ↓ RWC, ↓ membrane stability, ↑ LP, ↑ EL, ↑ SOD, POX, CAT, APX, GPX, and GR	60 mM	↑ growth, ↑ Chl, ↑ photosynthetic activity, ↑ *gS*, = *Ci*, ↑ number of stomata, = stomatal size, ↑ WUE, ↑ RWC, ↑ membrane stability, ↓ LP, ↓ EL, ↑ SOD, POX, CAT, APX, GPX, and GR	[141]
	Ni 77 mg kg^{-1} (Ni-rich soil)	*Triticum aestivum* L.	-	20 mM	↓ Ni uptake, ↑ photosynthetic rate, = *E*, = *gS*, = *Ci*, = LP, ↓ H2O2, = O2.-, ↑ SOD, = CAT, APX and DHAR, = grain protein, fiber, starch, polyphenols, and fat	[148]

	Pb 150-450 mg kg^{-1}	*Olea europaea* L.	↓ growth, ↑ H_2O_2, ↑ LP, ↑ EL, ↑ SOD, GPX, and APX, ↑ phenolics, ↑ flavonoids, ↑ $DPPH^•$ radical-scavenging activity	20 mM	↑ growth, ↓ Pb^{2+} uptake, ↓ H_2O_2, ↓ LP, ↓ EL, ↑ SOD, GPX, and APX, ↑ phenolics, ↑ flavonoids, ↑ $DPPH^•$ radical-scavenging activity	[145]
	Pb 500 mg kg^{-1}	*Phaseolus vulgaris* L.	↓ growth, ↓ Chl, ↓ RWC, ↑ LP, = SOD, CAT and APX	25-50 mM	↑ growth, ↑ Chl, ↑ RWC, ↓ Pb^{2+} uptake, ↓ LP, = SOD, CAT and APX	[214]
	Pb 7.5 mM	*Zea mays* L.	↓ growth, ↓ yield, ↓ Chl and Car, ↑ **proline** ↑ soluble sugars, ↑ LP, ↑ EL, ↑ GSH, ↑ AsA, ↑ free phenols, ↑ SOD, POX, and CAT, ↓ N, P, K^+ uptake	7.5 mM	↑ growth, ↑ yield, ↑ Chl, ↑ soluble sugars, ↓ LP, = EL, ↓ GSH, ↓ AsA, ↓ free phenols, ↓ SOD, POX, and CAT, ↑ N, P, K^+ uptake, ↓ Pb^{2+} uptake	[129]
	Pb 23959 mg kg^{-1} (Pb-polluted soil)	*Zea mays* L.	↓ Chl, ↑ Car, ↑ GB, ↑ LP, = phenolics, ↑ **proline**	5-10 mM	↑ Pb^{2+} uptake, ↑ Pb^{2+} efflux (at 10 mM), ↑ Chl (at 5 mM Pb), ↓ Chl (at 10 mM Pb), ↓ Car, ↓ LP (at 10 mM), ↓ GB, ↓ phenolics (at 5 mM Pb), ↑ phenolics (at 10 mM Pb)	[146]
	As 5-500 µM	*Oryza sativa* L.	↑ soluble proteins, ↓ free amino acids, ↑ **proline,** ↓ RNase, ↓ protease, ↓ leucine aminopeptidase, ↑ carboxypeptidase	1 M	↑ RNase	[215]
	As 5-25 µM	*Solanum melongena* L.	↓ growth, ↓ Chl, ↓ Chl fluorescence parameters, ↑ H_2O_2, ↑ O_2^-, ↑ LP, ↑ SOD, POX, and CAT, ↓ GST, ↑ **proline** ↑ P5CS, ↓ ProDH	25 µM	↑ growth, ↑ Chl, ↓ As uptake, ↑ yield of electron transport per trapped excitation, ↑ Φ PSII, ↑ performance index of PSII, ↓ H_2O_2, ↓ O_2^-, ↓ LP, ↑ SOD, POX, CAT, and GST, ↑ P5CS, ↓ ProDH	[142]
	As 75 µM	*Glycine max* L.	↓ growth, ↑ H_2O_2, ↑ O_2^-, ↑ LP, ↓ membrane stability, ↓ DNA content, ↑ DNA oxidation, ↑ DNase activity, ↑ DNA fragmentation, ↑ **proline**, ↓ genomic template stability, ↑ DNA polymorphism, ↓ SOD, CAT, and APX	10 mM	↑ growth, ↓ As uptake, ↓ H_2O_2, ↓ O_2^-, ↓ LP, ↑ membrane stability, ↑ DNA content, ↓ DNA oxidation, ↓ DNase, ↓ DNA fragmentation, ↑ genomic template stability, ↓ DNA polymorphism, ↑ SOD, CAT, and APX, ↑ P5CS	[134]

	Se<br>4-6 ppm	*Phaseolus vulgaris* L.	↓ growth, ↓ cellular respiration, ↓ Chl, ↓ leaf water content (at 6 ppm Se), ↑ EL, ↑ membrane damage, ↑ LP, ↑ H_2O_2, ↑ SOD, CAT and APX (at 4 ppm),<br>↓ APX and GR (at 6 ppm),<br>↓ AsA and GSH (at 6 ppm),<br>↓ **proline**	50 μM	↑ growth, ↑ Chl, ↑ cellular respiration, ↑ leaf water content (from 6 ppm Se), ↓ EL, ↓ LP, ↓ H_2O_2, ↓ membrane damage, ↑ SOD, CAT, APX, and GR, ↑ AsA and GSH	[143]
	Se<br>250 μM	*Nicotiana tabacum* L.	↓ biomass, ↑ cell death,<br>↑ intracellular ROS, ↑ SOD, CAT, APX, and POX	5-10 mM	↑ APX and POX, ↑ Se uptake	[135]
**Xenobiotics**	Basagran® herbicide (bentazone 48%)	*Trigonella foenum-graecum* L.	↓ root growth, ↓ shoot fresh and dry weight, = RWC,<br>↓ Chl, = Car,<br>↑ H_2O_2, ↑ LP, = EL,<br>= GSH, = TAC, ↑ **proline**, ↑ P5C, ↑ P5CS, ↓ **Proline** dehydrogenase (ProDH), ↑ CAT, ↑ GST	7 mM (seed treatment)	↑ growth, ↑ shoot and root fresh weight, = RWC,<br>= Chl, = Car, ↓ H_2O_2, ↓ LP, = EL, ↑ GSH, = TAC, ↓ **proline**, ↓ P5C, ↓ P5CS, ↑ ProDH, ↓ CAT, ↓ GST	[179]

↑, ↓, and = symbolize the reported increase, decrease or maintenance of a certain parameter, respectively.
ABA, abscisic acid; APX, ascorbate peroxidase; AsA, ascorbate; Asp, aspargine; BR, brassinosteroids; CA, carbonic anhydrase; Car, carotenoids; CAT, catalase; Chl, chlorophylls; Ci, intercellular CO2 concentration; CK, cytokinins; DHAR, dehydroascorbate reductase; DPPH, 2,2-diphenyl-1-picrylhydrazyl; E, transpiration rate; EL, electrolyte leakage; Fv/Fm, maximum quantum yield of PSII; GA3, gibberellic acid; GB, glycine betaine; GDH, glutamate dehydrogenase**;** Glu, glutamate; Gly I, glyoxalase I; Gly II, glyoxalase II; GOGAT, glutamate synthase; GPX, glutathione peroxidase; GR, glutathione reductase; gs stomatal conductance; GS, glutamine synthetase; GSH, glutathione; GSSG, glutathione disulfide; GST, glutathione S-transferase**;** GST, glutathione S-transferases; H2O2, hydrogen peroxide; IAA: indole acetic acid; JA, jasmonic acid; LP, lipid peroxidation; MDA, malondialdehyde; MDHAR, monodehydroascorbate reductase; NADPH, nicotinamide adenine dinucleotide phosphate; NPQ, non-photochemical quenching; NR, nitrate reductase; O2.-, superoxide anion; OAT, ornithine aminotransferase; P5C, pyrroline-5-carboxylate; P5CS, pyrroline-5-carboxylate synthase; PN, net photosynthetic rate; POX, peroxidase; PPO, polyphenol oxidase**;** ProDH, proline dehydrogenase; PTS, trisodium-8-hydroxy-1,3,6-pyrenetrisulphonic acid; Put, putrescine; ROS, reactive oxygen species; RuBisCO, ribulose-1,5-bisphosphate carboxylase-oxygenase; RWC, relative water content; SA, salicylic acid; SAMDC, S-adenosylmethionine decarboxylase; SOD, superoxide dismutase; Spd, spermidine; Spm, spermine; TAC, total antioxidant capacity; WUE, water use efficiency; Φ PSII, quantum yield of PSII.

Table S3

Effects on overall plant physiology and on the proline accumulation in plants facing heavy-metal-induced stress.

Stress factor		Plant species	Effects on overall plant physiology	Effects on the proline accumulation	Author notes	Citation
**Heavy metals**	Cu 1 - 1000 µM	*Silene vulgaris* (Moench) Garcke	↓ root growth, ↓ leaf water content	↑ **proline** accumulation in the leaves, no **proline** accumulation in the roots	**proline** accumulation related to the alleviation of water deficit, but not with the prevention of direct toxic effects of the HMs. Leaves of the tolerant ecotype did not show any accumulation of **proline** under Cu- or Zn-exposure, nor to Cd levels below 100 µM	[128]
	Cd 1 - 1000 µM					
	Zn 1 - 1000 µM					
	Cd 10-100 µg g^{-1}	*Solanum melongena* L.	↓ biomass, ↓ SOD, C in roots, ↓ shoot PCs synthesis, ↑ LP	no **proline** accumulation	Cd-induced oxidative stress occurs in *S. nigrum* without affecting growth; free **proline** accumulation and antioxidant defenses (but not PCs production) seem to play an important role against oxidative stress in Cd tolerance in the hyperaccumulator species	[126]
		Solanum nigrum L. (Cd-hyperaccumulator)	↑ root SOD, ↑ shoot POX, ↑CAT, ↑ accumulation of Cd in shoots, ↑ shoot PCs synthesis	↑ **proline** accumulation (mainly in the roots)		
	Ni 10-200 µM	*Triticum aestivum* L.	↓ growth, ↓ RWC, ↑ accumulation of Ni, ↑ H_2O_2, ↓ SOD, ↓ shoot CAT, ↑ shoot APX, POX and GST,	↑ **proline** accumulation (mainly in shoots)	Although Ni accumulation and growth reduction were less pronounced in the shoots, these organs were more responsive to Ni stress in terms of AOX activity and **proline** accumulation. Ni-stressed enhancement of **proline** might be associated with osmoprotective processes	[113]
	Pb 50-500 mg kg^{-1}	*Cymbopogon flexuosus* Stapf.	-	↑ **proline** accumulation (mainly in young leaves)	Cd is the strongest inducer of **proline** accumulation. **proline** accumulation can be used as a biochemical indicator for HM-stress in lemongrass. Over long term exposure to HMs, **proline** accumulation decreases	[108]
	Hg 50-500 mg kg^{-1}					
	Cd 50-200 mg kg^{-1}					

	Cd<br>50 - 400 µM	*Atriplex halimus* subsp. *schweinfurthii*	↓ growth, ↓ Chl, ↓ transpiration and root hydraulic conductivity, ↑ Cd uptake (mainly in the roots)	↑ **proline** accumulation (mainly in the roots)	*A. halimus* provides a new plant resource with potential for Cd hyperaccumulation	[124]
	Ni<br>50 mM	*Triticum aestivum* L.	↓ growth, ↓ Chl,<br>↓ CA,<br>↑ LP,<br>↑ EL, ↓ CAT	↑ **proline** accumulation	Enhanced **proline** levels improve osmotic adjustment, membrane stability and ROS scavenging activity. The production of **proline** was higher in plants co-exposed to GA_3 and Ca^{2+}, which explains the alleviation of Ni toxicity and reduction of oxidative damage in these plants	[115]
	Cr<br>0.4 mM	*Raphanus sativus* L.	↓ growth,<br>↓ Chl and Car,<br>↓ Hill reaction activity,<br>↑ marginal chlorosis,<br>↑ LP,<br>↑ sugars, ↓ protein,<br>↓ GR and CAT,<br>↓ uptake and translocation of Fe, S, P and Zn,<br>↑ Cr accumulation in the roots	↑ **proline** accumulation in leaves	-	[121]
	Ni<br>2 mM	*Glycine max* L.	↓ germination rate, ↓ growth, ↓ Fv/Fm, ↓ photochemical quenching, ↑ NPQ, ↑ GB, ↓ total protein, ↑ total soluble sugar, ↑ H2O2, ↑ LP, ↑ NADPH oxidase, ↑ SOD, CAT, APX, and POX, ↑ AsA	↑ **proline** accumulation	**proline** is a potential indicator of stress tolerance. **proline** accumulation was further induced by co-exposure to JA, possibly acting as an AOX to protect the cells from the oxidative burst	[116]
	Cr (III)<br>3, 12, 30 mg L^{-1}<br><br>Cr (VI)<br>1.6, 6.4, 12.8 mg L^{-1}	*Oryza sativa* L.	↑ Cr accumulation	↑ short-term **proline** accumulation in leaves and roots	**proline** from roots and shoots showed similar responses to Cr(III) exposure, but in response to Cr(VI). **proline** accumulation was more sensitive in shoots than in roots	[137]
	Cd<br>25 - 100 ppm	*Ricinus communis* L.	↓ growth,<br>↓ water content,<br>↓ Chl and Car,<br>↑ total phenolics	↑ **proline** accumulation in both leaves and roots	Free **proline** demonstrated positive correlations with Cd-accumulation, phenolics and dry biomass	[216]
	Ni<br>100 mM	*Solanum nigrum* L.	↓ biomass production, = root elongation, ↓ Chl, ↓ RuBisCO, ↑ Ni accumulation in roots, ↓ protein, ↓ H2O2, ↑ O2.- in roots, ↑ SOD, ↑ CAT in roots, ↑ APX in shoots	↑ **proline** accumulation (mainly in the roots)	In roots, increase of **proline** contributed to the enhancement of tolerance of *S. nigrum* plants to Ni	[125]

	Ni<br>10 - 200 µM	*Oryza sativa* L.	↓ growth, ↓ Chl, ↑ LP, ↑ H2O2, ↓ soluble proteins in shoots, ↓ soluble sugars, ↓ SOD, ↑ POX and CAT, ↑ AsA and GSH (mainly in the shoots)	↑ **proline** accumulation (mainly in the shoots)	**proline** is reported to increase as a result of increasing Ni stress	[121]
	Pb and Zn<br>25 - 100 mg kg^{-1}	*Raphanus sativus* L. and *Brassica oleracea* L.	↓ plant biomass, ↓ Chl and Car, ↑ phenolics in shoots	↑ **proline** accumulation in shoots	no visible symptoms of metal toxicity and high survival rates can be related to the increase of **proline**	[115]
	Ni<br>50 µM	*Solanum lycopersicum L.*	↓ growth, ↓ plant biomass,↑ H_2O_2, ↑ $O_2^{\cdot-}$ in shoots, ↑ GSH and AsA	↑ **proline** accumulation (mainly in the shoots)	**proline** accumulation resembled a signal of stress sensitivity	[122]

↑, ↓, and = symbolize the reported increase, decrease or maintenance of a certain parameter, respectively.
ABA, abscisic acid; APX, ascorbate peroxidase; AsA, ascorbate; Asp, aspargine; BR, brassinosteroids; CA, carbonic anhydrase; Car, carotenoids; CAT, catalase; Chl, chlorophylls; *Ci*, intercellular CO_2 concentration; CK, cytokinins; DHAR, dehydroascorbate reductase; DPPH, 2,2-diphenyl-1-picrylhydrazyl; *E*, transpiration rate; EL, electrolyte leakage; Fv/Fm, maximum quantum yield of PSII; GA_3, gibberellic acid; GB, glycine betaine; GDH, glutamate dehydrogenase**;** Glu, glutamate; Gly I, glyoxalase I; Gly II, glyoxalase II; GOGAT**,** glutamate synthase; GPX, glutathione peroxidase; GR, glutathione reductase; g_s stomatal conductance; GS, glutamine synthetase; GSH, glutathione; GSSG, glutathione disulfide; GST, glutathione S-transferase**;** GST, glutathione S-transferases; H_2O_2, hydrogen peroxide; IAA: indole acetic acid; JA, jasmonic acid; LP, lipid peroxidation; MDA, malondialdehyde; MDHAR, monodehydroascorbate reductase; NADPH, nicotinamide adenine dinucleotide phosphate; NPQ, non-photochemical quenching; NR, nitrate reductase; $O_2^{\cdot-}$, superoxide anion; OAT, ornithine aminotransferase; P5C, pyrroline-5-carboxylate; P5CS, pyrroline-5-carboxylate synthase; P_N, net photosynthetic rate; POX, peroxidase; PPO, polyphenol oxidase**;** ProDH, proline dehydrogenase; PTS, trisodium-8-hydroxy-1,3,6-pyrenetrisulphonic acid; Put, putrescine; ROS, reactive oxygen species; RuBisCO, ribulose-1,5-bisphosphate carboxylase-oxygenase; RWC, relative water content; SA, salicylic acid; SAMDC, S-adenosylmethionine decarboxylase; SOD, superoxide dismutase; Spd, spermidine; Spm, spermine; TAC, total antioxidant capacity; WUE, water use efficiency; Φ PSII, quantum yield of PSII.

Table S4

Effects of applying stress mitigators to plants facing xenobiotics-induced stress, namely on the endogenous levels of proline.

Stress factor		Plant species	Stress-induced effects in comparison to the control	Mitigation treatment	Treatment-induced effects in comparison to the stress factor	Reference
Xenobiotics	0.4 - 1.6 g ha^{-1} Clopyralid herbicide	*Brassica juncea* (L.) Czern.	↓ leaf water content, ↑ leaf temperature, ↓ Chl, ↓ Car, ↑ H_2O_2, ↑ LP, ↑ **proline** ↑ CAT, ↑ POD	Foliar spray or seed priming with SA (500 μM)	↑ leaf water content, ↓ leaf temperature, ↑ Chl, ↑ Car, ↓ H_2O_2, ↓ LP, ↓ **proline** ↓ CAT, ↓ POD	[216]
	30 mg kg^{-1} Glyphosate herbicide	*Hordeum vulgare* L.	↓ root growth, ↓ leaf and root fresh weight, = Chl, = Car, = relative RuBisco, ↑ leaf and root LP, ↑ leaf and root H_2O_2, = leaf $O_2^{\cdot-}$, ↑ root $O_2^{\cdot-}$ ↓ leaf AsA/DHA, ↓ leaf AsA/total AsA, ↑ leaf DHA/total AsA, = leaf total thiols, ↓ leaf protein thiols/total thiols, ↑ leaf non-protein thiols/total thiols, ↑ **leaf and root proline,** ↓ root total thiols, = root protein thiols/total thiols, = root non-protein thiols/total thiols, ↑ leaf and root SOD, ↑ leaf CAT, ↓ root CAT, ↑ leaf and root APX, ↑ leaf and root GST	Watering with SA (100 and 500 μM)	= root growth, = leaf and root fresh weight, = Chl, = Car, = relative RuBisco; = leaf and root LP, ↓ leaf and root H_2O_2, = leaf and root $O_2^{\cdot-}$, = leaf AsA/DHA, = leaf AsA/total AsA, = leaf DHA/total AsA, = leaf total thiols, ↓ leaf protein thiols/total thiols, ↑ leaf non-protein, thiols/total thiols, ↓ **leaf and root proline,** = root total thiols, = root protein thiols/total thiols, = root non-protein thiols/total thiols, ↑ leaf and root SOD, = leaf CAT, ↑ root CAT, = leaf APX, ↓ root APX, = leaf and root GST	[217]
	1500 g ha^{-1} Chlorothalonil fungicide	*Vitis vinifera* L.	= protein, **= proline**, ↑ LP, ↑ SOD, ↑ APX, ↓ CAT, ↑ POX, = AsA, = GSH, ↑ GR, ↑ GST, = *GSH,* = *GR,* = *GST,* = *MRP,* ↑ *P450,* ↑ Chlorothalonil residue	Foliar spray of 24-epibrassinolide (0.10-0.42 μM)	= protein, ↑ **proline**, = LP, = SOD, = APX, ↓ CAT, ↓ POX, = AsA, ↑ GSH, ↓ GR, ↑ GST, ↑ *GSH,* ↑ *GR,* ↑ *GST,* = *MRP,* = *P450,* ↓ Chlorothalonil residue	[183]
	0.5 mM Glyphosate herbicide	*Triticum aestivum* L.	↓ shoot and root length, ↓ shoot and root fresh weight, = H_2O_2, ↑ LP, ↑ **proline,** ↓ thiols, ↓ total phenols, ↑	Foliar spray of 24-epibrassinolide (1 μM)	↑ shoot length, = root length, ↑ shoot fresh weight, = root fresh weight, = H_2O_2, ↓ LP, **= proline**, = thiols, ↑ total phenols, ↓	[180]

			SOD, ↑ CAT, ↑ GPX, ↑ GR, ↑ GST		SOD, ↑ CAT, ↑ GPX, = GR, ↑ GST	
	400 mg kg $^{-1}$ Acetaminophen	*Hordeum vulgare* L.	= root length, = leaf fresh weight, ↓ leaf dry weight, ↓ root fresh and dry weight,<br>= Chl, = Car, ↑ leaf GS, = root GS, = leaf NR, ↓ root NR, = leaf and root LP, ↑ leaf $O_2^{\cdot-}$, = root $O_2^{\cdot-}$, = leaf H_2O_2, ↑ root H_2O_2, = leaf total thiols, ↑ root total thiols, = leaf protein-bond thiols, ↑ root protein-bond thiols,<br>**↑ leaf proline, = root proline**<br>= leaf total AsA, ↓ root total AsA, = leaf and root AsA/total AsA, = leaf and root DHA/total AsA, ↑ leaf SOD, = root SOD, ↓ leaf CAT, = leaf and root APX, ↑ leaf *HvAPX,* ↑ leaf *HvSOD,* ↑ leaf *HvCAT1,* = leaf *HvCAT2*	Silicon dioxide nanomaterial (nano-SiO_2; 3 mg kg $^{-1}$)	= root length, ↓ leaf fresh weight, = root fresh weight, = leaf and root dry weight,<br>= Chl, = Car, = leaf and root GS, ↓ leaf NR, ↑ root NR, = leaf and root LP, = leaf and root $O_2^{\cdot-}$, = leaf and root H_2O_2, ↑ leaf total thiols, = root total thiols, = leaf and root protein-bond thiols,<br>**= leaf proline, ↓ root proline,**<br>= leaf and root total AsA, = leaf and root AsA/total AsA, = leaf and root DHA/total AsA, = leaf and root SOD, = leaf CAT, = leaf APX, ↑ root APX, ↓ leaf *HvAPX,* ↓ leaf *HvSOD,* ↓ leaf *HvCAT1,* = leaf *HvCAT2*	[173]
	10 mg kg $^{-1}$ Glyphosate herbicide	*Solanum lycopersicum* L. cv. Micro-Tom	↓ root length, ↓ root and shoot fresh weight,<br>↑ shoot LP, ↓ root LP, ↑ shoot and root $O_2^{\cdot-}$,<br>= shoot and root H_2O_2<br>**↑ shoot and root proline**, ↑ shoot and root GSH, ↑ shoot AsA, = root AsA, ↑ shoot total AsA, = root total AsA, = shoot AsA/total AsA, ↑ root AsA/total AsA, = shoot and root DHA/total AsA, ↓ shoot AsA/DHA, ↑ root AsA/DHA, = shoot SOD, ↓ root SOD, ↓ shoot CAT and APX, ↓ root CAT and APX, = shoot and root GR, ↓ shoot DHAR, = root DHAR	Foliar spray of $Na_2SiO_3.5H_2O$ (1 mM) or nano-SiO_2 (1 mM)	↑ root length, ↑ root fresh weight, = shoot fresh weight, ↓ shoot LP, = root LP, = shoot $O_2^{\cdot-}$, ↓ root $O_2^{\cdot-}$, = shoot H_2O_2, ↓ root H_2O_2, ↓ **shoot and root proline,** = shoot GSH, ↓ root GSH, = shoot and root AsA, = shoot and root total AsA, = shoot and root AsA/total AsA = shoot and root DHA/total AsA, = shoot AsA/DHA, ↓ root AsA/DHA, = shoot SOD, ↑ root SOD, ↑ shoot CAT and APX, ↑ root CAT and APX, ↑ shoot GR, = root GR, ↑ shoot DHAR, = root DHAR, ↓ root glyphosate accumulation	[167]
	4 μM	*Oryza sativa* L.	↓ growth, = Chl, ↓ Car, ↑ LP, ↑ $O_2^{\cdot-}$, ↑	Seedling treatment with	↑ growth, ↑ Chl, ↑ Car, ↓ LP, ↓ $O_2^{\cdot-}$, ↓	[218]

	Butachlor herbicide		H_2O_2, ↑ **proline,** ↑ P5CS, ↓ ProDH, ↓ shoot protein, ↓ AsA, ↑ DHA, ↓ AsA/DHA, ↓ GSH, ↑ shoot GSSG, ↓ GSH/GSSG ↓ APX, GR, MDHAR and DHAR	Na_2SiO_3 (10 µM)	H_2O_2, ↓ **proline**, ↓ P5CS, ↑ ProDH, ↑ proteins, ↑ AsA, ↓ DHA, ↑ AsA/DHA, ↑ GSH, = GSSG, ↑ GSH/GSSG, ↑ APX, GR, MDHAR and DHAR	
	10 mg kg $^{-1}$ Glyphosate herbicide	*Solanum lycopersicum* L. cv. Micro-Tom	↓ root length, ↓ root and shoot fresh weight, ↑ shoot protein, ↓ root protein, ↓ shoot NR, = root NR, ↑ shoot LP, ↓ root LP, = shoot and root H_2O_2, ↑ shoot and root $O_2^{\cdot -}$, ↑ **shoot and root proline**, = shoot and root total AsA, = shoot and root AsA/DHA, ↑ shoot and root GSH, = shoot TAC, ↓ root TAC, ↓ shoot total phenols, = root total phenols, ↓ shoot flavonoids, = root flavonoids, = shoot SOD, ↓ root SOD, ↓ shoot GST, ↓ root GST, ↓ shoot and root APX and CAT	Sodium nitroprusside (200 µM)	= root length, ↑ root fresh weight, = shoot fresh weight, = shoot protein, ↑ root protein, = shoot and root NR, = shoot and root LP, = shoot H_2O_2, ↓ root H_2O_2, ↓ shoot and root $O_2^{\cdot -}$, ↓ **shoot and root proline,** = shoot and root total AsA, = shoot and root AsA/DHA, = shoot GSH, ↓ root GSH, = shoot and root TAC, = shoot and root total phenols, = shoot and root flavonoids, = shoot SOD, ↑ root SOD, ↑ shoot GST, = root GST, ↑ shoot and root APX and CAT	[168]

↑, ↓, and = symbolize the reported increase, decrease or maintenance of a certain parameter, respectively.
ABA, abscisic acid; APX, ascorbate peroxidase; AsA, ascorbate; Asp, aspargine; BR, brassinosteroids; CA, carbonic anhydrase; Car, carotenoids; CAT, catalase; Chl, chlorophylls; *Ci*, intercellular CO_2 concentration; CK, cytokinins; DHAR, dehydroascorbate reductase; DPPH, 2,2-diphenyl-1-picrylhydrazyl; *E*, transpiration rate; EL, electrolyte leakage; Fv/Fm, maximum quantum yield of PSII; GA_3, gibberellic acid; GB, glycine betaine; GDH, glutamate dehydrogenase**;** Glu, glutamate; Gly I, glyoxalase I; Gly II, glyoxalase II; GOGAT**,** glutamate synthase; GPX, glutathione peroxidase; GR, glutathione reductase; g_s stomatal conductance; GS, glutamine synthetase; GSH, glutathione; GSSG, glutathione disulfide; GST, glutathione S-transferase**;** GST, glutathione S-transferases; H_2O_2, hydrogen peroxide; IAA: indole acetic acid; JA, jasmonic acid; LP, lipid peroxidation; MDA, malondialdehyde; MDHAR, monodehydroascorbate reductase; NADPH, nicotinamide adenine dinucleotide phosphate; NPQ, non-photochemical quenching; NR, nitrate reductase; $O_2^{\cdot -}$, superoxide anion; OAT, ornithine aminotransferase; P5C, pyrroline-5-carboxylate; P5CS, pyrroline-5-carboxylate synthase; P_N, net photosynthetic rate; POX, peroxidase; PPO, polyphenol oxidase**;** ProDH, proline dehydrogenase; PTS, trisodium-8-hydroxy-1,3,6-pyrenetrisulphonic acid; Put, putrescine; ROS, reactive oxygen species; RuBisCO, ribulose-1,5-bisphosphate carboxylase-oxygenase; RWC, relative water content; SA, salicylic acid; SAMDC, S-adenosylmethionine decarboxylase; SOD, superoxide dismutase; Spd, spermidine; Spm, spermine; TAC, total antioxidant capacity; WUE, water use efficiency; Φ PSII, quantum yield of PSII.
